# Orthodontic apps: An assessment of content accuracy and validity

**DOI:** 10.1177/14653125221131064

**Published:** 2022-10-15

**Authors:** Dharshini Prithiviraj, Robert SD Smyth, Mohammad Owaise Sharif

**Affiliations:** 1 Orthodontic Department, UCL Eastman Dental Institute, London, UK; 2 Orthodontic Department, Glan Clwyd Hospital, Rhyl, UK

**Keywords:** orthodontic apps, mobile apps, content accuracy, smartphones

## Abstract

**Objective:**

To assess the content accuracy of orthodontic treatment information in patient-focused apps.

**Design:**

A cross-sectional review study.

**Setting:**

Orthodontic apps available on the UK Android and Apple App Stores.

**Methods:**

Apps identified in a previous research study and those identified via a questionnaire of specialist orthodontists were assessed for accuracy of content utilising an evidence-based checklist. The checklist covered five main orthodontically relevant themes and 32 codes with respective items.

**Results:**

The accuracy of information content for 16 patient-focused apps was assessed. Eight apps provided information related to orthodontic treatment and handling emergencies. Five apps were reminder apps and a small number (n = 3) contained games and timers for toothbrushing and aligners. With regard to the accuracy of information content, only two apps contained information across all five themes of the evidence-based checklist. Only one app received a score of ‘fair - excellent’ under the oral hygiene theme; interestingly, this app was the most commonly used patient-focused app. Eight apps containing orthodontic treatment information scored poorly as they had inaccurate information on handling emergency situations. None of the apps were deemed excellent with regard to accuracy of information content.

**Conclusion:**

The orthodontic mobile apps assessed in this study mostly contained information of poor accuracy. Therefore, there is a need for high-quality apps with credible information supported by evidence to be developed.

## Background

In recent years, there has been a proliferation of mobile apps and orthodontic patients have expressed their willingness to use an orthodontic app that would aid in treatment (Gupta and Vaid, 2017; Sharif et al., 2019). Patient-focused apps are those that may help improve the patient experience with regard to accessing health information, clinician-to-patient communication, feedback and monitoring. This in turn may aid effective compliance and behaviour modifications, as these approaches address the various components of the COM-B model of the Behaviour Change Wheel, as described by Michie et al. (2011). In orthodontics, clinicians often want to generate a behaviour change in their patients in order to improve compliance, and provision of information and improvement of knowledge is integral to this. However, an individual’s Capability, Opportunity and Motivation may also need to be altered to generate a behaviour change. The interplay of these factors is summarized in the COM-B model.

Previous research has highlighted that the information content of dental and orthodontic apps is unsatisfactory (Sharif and Alkadhimi, 2019; Siddiqui et al., 2021; Tiffany et al., 2018). An assessment of the quality of orthodontic apps using the Mobile App Rating Scale (MARS) and behaviour change techniques by Siddiqui et al. (2021) found that there was currently a very limited number of orthodontic apps of sufficient quality to recommend to patients. This study mainly focused on assessing the functionality of apps using the MARS tool. A study by Sharif and Alkadhimi (2019) assessed the quality of oral hygiene instructions in apps using an evidence-based checklist and highlighted the need for improvement in information content. Currently, there appears to be a limited number of studies that have assessed the content accuracy of apps including a wider range of orthodontic themes (Rao et al., 2018; Singh, 2013). However, the pool of available apps is constantly evolving, and it is important that professionals keep abreast of the latest developments and app releases.

For healthcare professionals, mobile apps may help to simplify practice administration, including patient records and communication, alongside practice development and continuing professional development (Ventola, 2014). A recent scoping review of consumer-facing apps found that the content of many apps was not based on the available evidence or indeed may have contained information that contradicted the best available evidence (Akbar et al., 2019).

The aim of the present study was to analyse the content accuracy of generic information provided on these apps to ensure safe clinical practice.

## Methods

This study was part of a broader research project that took part in two stages.

Stage 1: Questionnaire development and distribution (via University College London [UCL] OPINIO software) to consultant orthodontist and specialist orthodontist groups of the British Orthodontic Society (BOS). The questionnaire aimed to assess the awareness and use of mobile apps for patient information and practice development (Prithiviraj et al., 2022).
Stage 2: Identification of patient-focused apps. Those apps identified in a previous research study by Siddiqui et al. (2019) and those identified via Stage 1 available on the UK Android and Apple App Stores were assessed for accuracy of content, which forms the basis of this paper.

### Ethical considerations

Ethical approval was granted by the UCL Research Ethics Committee on 7 November 2019 (Project ID/Title: 16177/001). Clinical governance approval from the BOS was also requested to allow questionnaire distribution; this was granted on 16 January 2020.

### Development of an evidence-based checklist

An evidence-based checklist was created to help with content analysis. This was derived from peer-reviewed resources including the BOS advice sheets and information leaflets (British Orthodontic Society, 2012, 2014a–g), National Institute of Clinical Excellence guidelines, Public Health England’s ‘Delivering Better Oral Health: An evidence-based toolkit for prevention’ (Public Health England, 2017) and Cochrane reviews. Most of the information was obtained from the ‘Delivering Better Oral Health’ toolkit and BOS leaflets as they matched the information regularly given to orthodontic patients. Some information in the checklist, especially on handling emergency situations, was purely based on clinical practice and was denoted by an asterisk (*). The checklist covered five main orthodontically relevant themes and 32 codes with respective items (Table 1). The themes included were: oral hygiene; dietary advice; fixed appliances; orthodontic retention; and emergency situations.

**Table 1.** Evidence-based checklist for content analysis.

	1. Oral hygiene		2. Dietary advice		3. Fixed appliances		4. Orthodontic retention		5. Emergency situations	
	Code	Item	Code	Item	Code	Item	Code	Item	Code	Item
A	Type of toothbrush	Manual or powered toothbrush. Small toothbrush head, medium texture	Sugar intake	The frequency and amount of consumption of sugars should be reduced and limited to mealtimes	Duration of treatment	12–30 months, but this will vary according to how severe the tooth problem is. Missed appointments or repeated breakages of the brace will add to overall treatment time	What is a retainer?	Retainers are designed to keep your teeth straight and it is important to wear them as instructed	Emergency situations	If there is a part of the brace that has become loose or broken, remove it and keep it aside
B	Brushing frequency	Brush at least twice daily, for a minimum of 2 min, with a fluoridated toothpaste	When to avoid sugar	Avoid sugary foods and drinks between mealtimes and at bedtimes. Avoid foods and drinks containing sugar at bedtime when saliva flow is reduced and buffering capacity is lost	Frequency of appointments	Appointments will be scheduled regularly (usually 5–8 weeks) for the brace to be adjusted	Types of retainer	Retainers may either be removable or fixed and the orthodontist will advise which retainer is needed		Do not try and use any tools or instruments to remove it by yourself
C	Brushing time	Brush last thing at night and at least on one other occasion	Types of cariogenic food and drinks	Examples include sugared soft drinks; sweets, chocolate, confectionery, cakes and biscuits; table sugar; breakfast cereals, dried fruits, jams, preserves, honey, fruit in syrup or canned in juice; fresh fruit juices (one 150-mL glass of fresh fruit juice can count towards ‘five a day’); sugared, milk-based beverages and sugar-containing alcoholic drinks	Oral hygiene	It is important to brush your teeth for at least 2 min twice a day with a fluoride toothpaste. Interdental brushes may help clean around the brace and in between the teeth. A fluoride mouthwash should also be used daily, at a different time of the day to when you brush your teeth	Changes to expect with retainers	Speech alteration – temporary Increase in production of saliva –temporary		Pay a visit to a casualty clinic as soon as possible to prevent any potential injury
D	Use of fluoride	Use fluoridated toothpaste with at least 1350 ppm fluoride in adults and 1350–1500 ppm in children aged >7 years and young adults			Diet	Avoid sugary snacks/drinks between meals and at bedtime. Avoid sticky, chewy or hard sweets, mints and sugared chewing gum. Avoid fizzy drinks and large amounts of fruit juice. Avoid hard or chewy foods, such as apples, carrots and crusty bread, or cut them up first as these can damage your brace	Retention period	Indefinitely on a part-time basis		If any part of the brace is accidentally swallowed, go to the A&E immediately and report this to your orthodontist when you see them next (clinically accepted*)
E	Rinsing	Spit out after brushing and do not rinse			Analgesics	It is likely to be sore for about 3–5 days each time the brace is adjusted. If necessary, simple painkillers, such as the ones you would normally take for a headache, should help	Changes to expect if retainers are not worn as instructed	It is likely that if the patient stops wearing retainers there will be some tooth movement. Changes in the position of the teeth can continue throughout life and are part of the normal ageing process		
F	Interdental cleaning	Around orthodontic appliances and bridges: use kit suggested by the dental professional, which may include dental floss, tepes, interdental or single tufted brushes			Emergencies	Ring for an appointment as soon as is reasonably possible	Removal of retainers	Retainers should only be removed for eating, cleaning, contact sports and swimming		
G	Fluoride mouthwash use	Use a fluoride mouth rinse daily (0.05% NaF) at a different time from brushing			Use of wax	If the brace rubs your lips or cheeks, you can use some dental wax to help with this	Eating and drinking with retainers	Avoid sugary snacks/drinks between meals and at bedtime. Avoid sticky, chewy or hard sweets, mints and sugared chewing gum. Avoid fizzy drinks (including diet drinks) and large amounts of fruit juice. Hard or chewy foods, such as apples, carrots and crusty bread, can damage your retainer. Avoid them or cut them up first		
H					Benefits of fixed appliances	Removal of dental crowding or closing spaces. Alignment of upper and lower dental arches. Correction of the bite so the front and back teeth meet together evenly on closing. Reducing the likelihood of damage to prominent teeth. Enhancing facial aesthetics. Accommodating impacted, unerupted or displaced teeth. Preparation for advanced dental treatment such as crowns, bridges or dental implants	Storage of retainers	Retainers should be stored in a box when worn part-time		
I					Risks associated with orthodontic treatment	Pain and discomfort. Marks and stains on teeth due to poor oral hygiene. Shortening of roots. Gums can get puffy, sore and start to bleed if oral hygiene is poor. Poor compliance will prevent achieving good results	Cleaning retainers	Gently clean the retainer with a toothbrush and cold water over a sink, taking care not to drop it or use a retainer cleaning solution		
J					Playing wind instruments	Treatment with a fixed orthodontic appliance may cause a disruption to the playing ability of wind instrumentalists. The patient should seek advice from the teacher about the most appropriate time to commence orthodontic treatment and to avoid orthodontic adjustment appointments before important musical examinations or auditions	Retainer reviews	These are important and should occur regularly		
K							Repair and replacement of retainers	Ring for an appointment as soon as possible if retainers need repair or replacement		

The apps were given a score on a 4-point Likert-type scale in each category, based on the content available within them. The scoring system was as follows: 1 = information not present; 2 = information present, not accurate; 3 = information present, incomplete (i.e. no inaccurate information, but the information present was incomplete, for example, stating that retainers should be worn, but failing to provide the recommended wear period); 4 = information present and accurate; and N/A = apps that were not designed to hold information for a particular theme were scored as N/A.

The checklist was piloted by members of the research team. Five mobile apps were initially reviewed and scored using the checklist to assess feasibility. Results were interpreted in the form of tables and graphs and discussed by the research team. The checklist was updated based on the feedback received and changes were made to the scoring scheme, taking into consideration that some of the apps were not designed to provide information on any of the relevant themes from the checklist. These were coded as not applicable (N/A) to differentiate the score from information that should have been present and correct but which was not included. One author (MOS) has experience of developing checklists similar to the one included in this paper and has published related research in the past (Sharif and Alkadhimi, 2019; Smyth et al., 2019).

An average score per app was subsequently calculated using eligible themes. The following scoring scale was subsequently used to correlate with accuracy of content: 1 and 2 = poor; 3 = fair; and 4 = excellent.

### Identification of apps

A total of 18 apps were previously identified and were obtained from the Apple and Android App stores (Siddiqui et al., 2019). The apps were downloaded for content analysis where possible, but it was noted that some of the apps (n = 4) were removed and no longer accessible. Therefore, only 14 out of those 18 apps were used for this study.

**Figure 1.**
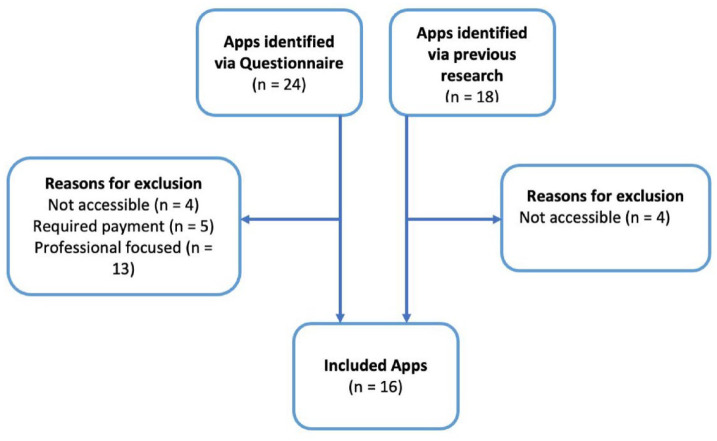
Flow chart of included apps.

A number of additional apps were also identified from the questionnaire detailed in Stage 1 (n = 24) and they were classified into patient-focused and profession-focused apps. Four apps were excluded as they were not available at the time of the study and five apps were not accessible as they required a log in. Therefore, the total number of apps from the questionnaire that were investigated was 15. From these 15 apps, two apps were finally included for content analysis after excluding apps that were profession-focused, needed logins, unavailability on the app store and repetitions from those already identified. Figure 1 shows that 16 apps in total were included in this study, which included 14 apps identified in previous research and two apps from the questionnaire (Siddiqui et al., 2019).

A content analysis of all included apps was carried out using the developed evidence-based checklist to assess their accuracy and validity. Descriptive statistics are presented.

## Results

### Assessment of generic knowledge content of apps

The accuracy of information content for the 16 patient-focused apps was assessed. Eight apps provided generic information on orthodontic treatment and handling emergencies. Five apps were reminder apps and the remainder (n = 3) contained games and timers for toothbrushing and aligners.

### Mean scores per app

Table 2 shows the mean accuracy score of the apps assessed. Apps that scored N/A for all themes were not included in the table (apps 3, 5, 8, 12 and 13). Apps 10 and 15 received a ‘fair’ average score across that particular app’s included themes. Apps 9, 11 and 14 received an average score that was in the range of ‘poor - fair’. All remaining apps (n = 6) obtained a ‘poor’ average score across all the included themes.

**Table 2.** Mean scores per theme for individual apps.

App	1. Oral hygiene	2. Dietary advice	3. Fixed appliances	4. Orthodontic retention	5. Emergency situations	Average score per app	Overall app accuracy
1	3	3	3	1	2	2.4	Poor
2	N/A	3	3	1	2	2.25	Poor
4	3	3	3	1	2	2.4	Poor
6	3	N/A	3	1	2	2.25	Poor
7	N/A	N/A	N/A	N/A	2	2	Poor
9	N/A	3	3	3	2	2.75	Poor - fair
10	N/A	N/A	N/A	3	N/A	3	Fair
11	N/A	3	3	3	2	2.75	Poor - fair
14	N/A	N/A	3	3	2	2.66	Poor - fair
15	3	3	N/A	N/A	N/A	3	Fair
16	1	N/A	N/A	N/A	N/A	1	Poor

### Mean scores of apps across each theme

Table 3 shows the mean scores for the apps assessed per theme. The dietary advice and fixed appliance themes obtained a ‘fair’ accuracy score when all the apps were considered. The oral hygiene theme received a mean score which was in the range of ‘poor - fair’. The remaining themes obtained a ‘poor’ mean score.

**Table 3.** Mean scores and overall accuracy per theme.

	1. Oral hygiene	2. Dietary advice	3. Fixed appliances	4. Orthodontic retention	5. Emergency situations
Mean score per theme	2.6	3	3	2	2
Overall accuracy per theme	Poor - fair	Fair	Fair	Poor	Poor

### App scores per theme

#### Oral hygiene

Table 4 shows that out of 16 apps, only five contained an oral hygiene section (apps 1, 4, 6, 15 and 16). App 1 obtained a score that was in the range of ‘poor - fair’. App 4 was deemed to have ‘poor’ accuracy of content for most of the items in this section, as it had no information on types of toothbrush, brushing frequency and time, use of fluoride and rinsing instructions; however, it obtained an ‘excellent’ score for one item as it had accurate information on interdental cleaning. App 6 was deemed ‘excellent’ for two items (rinsing and interdental cleaning) and ‘fair’ for two items as it had some information on the use of fluoride mouthwash and toothpaste. However, it obtained a ‘poor’ score for the remaining three items in this theme. App 15 performed the best under this theme by scoring ‘excellent’ for all items except for one item, which was interdental cleaning, where it received a ‘fair’ score. App 16 scored ‘poor’ for each of the items. Apps 2, 3, 5 and 7–14 were scored as N/A on all items.

**Table 4.** Oral hygiene scores.

App	1A: Type of toothbrush	1B: Brushing frequency	1C: Brushing time	1D: Use of fluoride	1E: Rinsing	1F: Interdental cleaning	1G: Use of fluoride mouthwash	Mean score	Overall app accuracy
1	1	3	1	3	3	4	3	2.57	Poor - fair
4	1	1	1	1	1	4	1	1.42	Poor
6	1	1	1	3	4	4	3	2.42	Poor
15	4	4	4	4	4	3	4	3.85	Fair - excellent
16	1	1	1	1	1	1	1	1.0	Poor

#### Dietary advice

Table 5 shows six out of 16 apps contained dietary advice (apps 1, 2, 4, 9, 11 and 15), with three apps (2, 4 and 9) being deemed ‘excellent’ for one item (present and accurate information on types of cariogenic food and drinks). App 15 was deemed ‘excellent’ for one item (present and accurate information on frequency of sugar intake). Apps 3, 5–8, 10, 12–14 and 16 were scored as N/A.

**Table 5.** Dietary advice scores.

App	2A: Sugar intake	2B: When to avoid sugar	2C: Types of cariogenic food and drinks	Mean score	Overall app accuracy
1	3	1	3	2.33	Poor
2	1	1	4	2.0	Poor
4	1	1	4	2.0	Poor
9	1	1	4	2.0	Poor
11	1	1	3	1.66	Poor
15	4	1	1	2.0	Poor

#### Fixed appliances

Out of 16 apps, seven contained advice relating to fixed appliances (Table 6). App 1 scored ‘excellent’ for two items (accurate information on emergencies and use of dental wax). A ‘fair’ score was given for another four items as the app touched upon some information on oral hygiene, diet for fixed appliance patients, use of analgesics and benefits of treatment. It scored poorly for the remaining items due to lack of information on treatment duration, appointment frequencies, risks and instructions on wind instruments. Apps 2, 4, 6, 9 and 11 had a similar spread of scores with a slightly different profile of information. App number 14 was deemed ‘excellent’ for only one item and scored poorly for all other items in this theme. None of the apps had information on duration of treatment, risks and wind instruments. Apps 3, 5, 7, 8, 10, 12, 13, 15 and 16 were scored as N/A on all items.

**Table 6.** Fixed appliance scores.

App	3A: Duration of treatment	3B: Frequency of appointments	3C: Oral hygiene	3D: Diet	3E: Analgesics	3F: Emergencies	3G: Use of wax	3H: Benefits of fixed appliances	3I: Risks associated with orthodontic treatment	3J: Playing wind instruments	Mean score	Overall app accuracy
1	1	1	3	3	3	4	4	3	1	1	2.4	Poor
2	1	1	1	3	4	1	4	3	1	1	2.0	Poor
4	1	1	3	3	4	1	4	1	1	1	2.0	Poor
6	1	1	3	1	4	4	4	3	1	1	2.3	Poor
9	1	1	1	4	4	1	4	1	1	1	1.9	Poor
11	1	3	1	3	3	4	1	3	1	1	2.1	Poor
14	1	1	1	1	1	1	4	1	1	1	1.3	Poor

#### Orthodontic retention

Eight apps (Table 7) were scored for retention content. App 9 obtained an ‘excellent’ score for having information on types of retainers and repairs and a ‘fair’ score for 1 item, which was on diet advice but had no other information on any of the other items such as changes to expect, retention period, cleaning, storage, reviews, etc. Similarly, app 10 received an ‘excellent’ score for having information on what retainers are but scored poorly for all other items due to lack of information. App 14 received the highest score as it was deemed excellent for having information on retainers, retainer types and the importance of retainer reviews but scored poorly for the other items due to inaccurate information on retainer wear and lack of generic information. Five apps had no information on orthodontic retention and scored poorly for all items.

**Table 7.** Orthodontic retention scores.

App	4A: What is a retainer?	4B: Types of retainer	4C: Changes to expect with retainers	4D: Retention period	4E: Changes to expect if retainers are not worn as instructed	4F: Removal of retainers	4G: Eating and drinking with retainers	4H: Storage of retainers	4I: Cleaning retainers	4J: Retainer reviews	4K: Repair and replacement of retainers	Mean scores	Overall app accuracy
1	1	1	1	1	1	1	1	1	1	1	1	1.0	Poor
2	1	1	1	1	1	1	1	1	1	1	1	1.0	Poor
4	1	1	1	1	1	1	1	1	1	1	1	1.0	Poor
6	1	1	1	1	1	1	1	1	1	1	1	1.0	Poor
9	1	4	1	1	1	1	3	1	1	1	4	1.45	Poor
10	4	1	1	1	1	1	1	1	1	1	1	1.27	Poor
11	1	1	1	1	1	1	1	1	1	1	1	1.0	Poor
14	4	4	1	2	1	1	1	1	1	4	1	1.9	Poor

#### Emergency situations

Table 8 shows that 50% of the apps (n = 8) contained information relating to emergency situations and obtained a ‘poor’ score, indicating that information was provided but was not entirely accurate. All other apps (apps 3, 5, 8, 10, 12, 13, 15 and 16) scored N/A on all items.

**Table 8.** Emergency situations scores.

App	5: Emergency situations	Overall app accuracy
1	2	Poor
2	2	Poor
4	2	Poor
6	2	Poor
7	2	Poor
9	2	Poor
11	2	Poor
14	2	Poor

## Discussion

### Assessing the quality of apps

The content analysis of apps in this study was carried out using an all-inclusive evidence-based checklist. The checklist covered five major themes that are significant and relevant to orthodontic patients in terms of knowledge content. The themes included oral hygiene, dietary advice, fixed appliances, orthodontic retention and emergency situations, which are the main areas patients are advised on when undergoing orthodontic treatment. The checklist was created after referring to peer-reviewed resources such as BOS advice sheets and leaflets and Public Health England’s ‘Delivering Better Oral Health: An evidence-based toolkit for prevention’ (British Orthodontic Society, 2012, 2014a–g; Public Health England, 2017). The majority of information was obtained from these sources as it was similar to the information that is routinely given to orthodontic patients in an NHS practice. The evidence-based checklist explored all significant areas of orthodontic treatment and highlighted important information that apps should contain to support orthodontic patients throughout their treatment process and to also educate them on dental health and appliance care and as such was a robust method of assessing the content accuracy of apps.

From the results obtained, it was evident that only a very small number of apps (n = 2) had information relating to all themes. Both apps 1 and 4 had some information on all themes but scored ‘poor’ overall due to the inaccuracy of information provided. The dietary advice and fixed appliances themes obtained a ‘fair’ mean score by all the apps that scored under them. The oral hygiene theme received a mean score which was in the range of ‘poor - fair’. The remaining themes received a mean score of ‘poor’ by all the apps that scored under them.

### Assessment of the methodology

The methodology used in this study was compared to that used by Sharif and Alkadhimi (2019), who conducted a study on the assessment of quality and knowledge content of patient-focused oral hygiene apps. The quality assessment was performed using the MARS tool and knowledge content of apps was assessed using an eight-item evidence-based checklist for oral hygiene. Apart from information quality, the apps were also assessed for engagement, functionality and aesthetics. A total of 20 apps were assessed from both the Apple and Google Play stores. In comparison, the checklist used in this study was a more extensive checklist that included five different themes such as oral hygiene, dietary advice, fixed appliances, orthodontic retention and emergency situations, with several codes and items pertaining to each theme. This allowed for a more thorough analysis of content but may also have led to poor scoring of some apps for not holding information for each item under a particular theme. Another study by Meade et al. (2020) looked at the quality of information provided by dental professionals on orthodontic retention and retainers on YouTube. The study used a similar methodology where a 4-point scoring system was used to score the quality of information in 10 predetermined domains. The domains were selected from evidence-based resources. A total of 62 YouTube videos were finally included in the study. The study concluded that the quality of information on orthodontic retention and retainers provided by dental professionals on YouTube was poor. As the checklist used in this study was meant for only orthodontic retention, the final scores of the videos gave a clearer understanding of the content quality pertaining to one particular theme.

### Commonly used apps

App 15 received an ‘excellent’ score for six items and a ‘fair’ score for one item in the oral hygiene theme, which meant it had an overall score of ‘fair - excellent’. This app was purely an oral hygiene app and was not expected to contain any orthodontic advice except for the oral hygiene aspect of it. The app helps patients set reminders for toothbrushing and appointments. Patients are able to listen to their favourite song while brushing to help keep them engaged. The app is also supplemented with animated videos on toothbrushing, interdental cleaning and flossing, making it an app with good functionality and aesthetics. A small amount of dietary advice is also included in this app. This could be the reason why this app was mentioned by 39% of the respondents as the commonly used patient-focused app in the questionnaire aspect of Stage 1 of this study (Prithiviraj et al., 2022).

### Lack of information available in apps

Apps that contained information on fixed appliances had no content on the potential duration of treatment and risks involved. This is important to incorporate into future apps as it is a significant part of treatment that patients should be aware of and consented for. The lack of information on orthodontic retention is concerning as apps on fixed appliances are expected to also have content on retention. This is especially important as patients are likely to forget about retainers and the importance of retention by the end of their orthodontic treatment. Apps may allow patients to understand retention better with the help of pictures and illustrations.

Of the apps, 50% (n = 8) scored poorly under the emergency situations theme as they provided inaccurate information (e.g. using a nail clipper to cut a long arch wire). This can lead to soft tissue injuries, risk of ingesting loose objects and further damage to the appliance, which may be detrimental or harmful to patients. Six apps also had features that allowed patients to send pictures to their clinicians in case of emergencies. This may be beneficial for getting instant advice but may not be the case for every situation. It would be helpful to have further research on patients’ and clinicians’ perceptions on using this feature to handle emergency situations.

### Implications for future research

The quality of the patient-focused apps currently available appears to be very low, highlighting the need for more credible, evidence-based apps that can be recommended for patients. This was also evident in a study by Tiffany et al. (2018). The authors assessed the content and usability of some popular and highly rated oral health promotion apps. The study showed that out of 33 apps that were reviewed, 67% were generated for the general public and not just dental patients. Of the apps, 58% were sponsored by software developers and not oral health experts, thereby lacking any theoretical basis for the content and were not validated. Of the apps, 58% also contained some educational content to encourage better oral health behaviour such as reminders for brushing and appointments, but overall the apps performed poorly in terms of content and also usability. It is apparent that there is a need for high-quality, evidence-based orthodontic apps to be developed with the objective that these may be utilised to improve patients’ compliance with treatment.

### Implications for practice

While there are clearly apps available that are good for certain aspects of a patient’s treatment journey, clinicians will ultimately have to consider recommending several different apps, YouTube videos and traditional paper leaflets for information delivery to improve compliance. Previous research has shown that there is a lack of high-quality YouTube videos relating to oral hygiene instruction and caution should be given in recommending these to patients (Smyth et al., 2019). It is apparent, however, that there is a need for high-quality, evidence-based orthodontic apps to be developed with the objective that these may be utilised to improve patients’ compliance with treatment alongside other methods of information delivery.

### Study limitations

In this study on mobile apps, the apps were only assessed for knowledge content and not usability. All the apps included were directed only to orthodontic patients and not the general public. As some of the apps contained information that lacked evidence or a strong theoretical basis, it is possible that they may have also been developed by software developers and not oral health experts. Several apps did appear to serve as good reminder apps. In terms of overall knowledge content, none of the apps were deemed excellent with regards to accuracy.

## Conclusion

A content analysis of 16 apps that were identified previously by members of the research team and patient-focused apps identified from the questionnaire was carried out. Only two out of 16 apps contained information across all five themes of an evidence-based checklist. Eight apps scored poorly for containing inaccurate information on handling emergency situations. None of the apps were deemed excellent in terms of accuracy of content. There is therefore a need for high-quality and evidence-based orthodontic apps to be created, which may be utilised to improve patients’ compliance with treatment.
